# Paraspinal Compartment Syndrome Associated with Opioid Overdose: A Case Report

**DOI:** 10.5811/cpcem.42227

**Published:** 2025-08-13

**Authors:** Terrence Habiyaremye, Kelly Holz, Jessica R. Jackson

**Affiliations:** Lewis Katz School of Medicine at Temple University, Department of Emergency Medicine, Philadelphia, Pennsylvania

**Keywords:** fentanyl, xylazine, paraspinal compartment syndrome

## Abstract

**Introduction:**

Compartment syndrome is an emergent condition of increased pressure within a muscle compartment. The paraspinal region is a rare location for compartment syndrome and is typically secondary to exertion, trauma, or surgery.

**Case report:**

We present a case of paraspinal compartment syndrome in a patient who presented after fentanyl overdose. This patient was likely exposed to xylazine, also known as “tranq,” which may have contributed to his presentation.

**Conclusion:**

Emergency physicians must be aware of paraspinal compartment syndrome to facilitate rapid diagnosis and treatment and prevent associated morbidity and mortality.

## INTRODUCTION

Compartment syndrome is an emergent condition of increased pressure within a muscle compartment; it most commonly results from trauma and typically affects the distal extremities. The paraspinal compartments are an uncommon anatomic location for this surgical emergency, for which the literature is limited. Delay in recognition of this condition and subsequent consultation for emergent surgical intervention carries the potential for ischemia, necrosis, and organ damage with ultimate poor health outcomes and quality of life. In existing case reports and reviews for acute paraspinal compartment syndrome, the etiology of this patient presentation is typically from exertion, trauma, or surgery.^1^ However, other recognized causes of compartment syndrome include prolonged immobilization,^2^ soft tissue infection,^3^ non-traumatic muscle injury,^4^ and spontaneous hemorrhage and hematoma.^5^ Our case report highlights a unique situation that proposes an additional risk factor: prolonged immobilization secondary to fentanyl use with likely xylazine adulteration.

## CASE REPORT

A 39-year-old man with a past medical history of anxiety, depression, hepatitis C, prior deep vein thrombosis, and polysubstance use disorder presented to the emergency department (ED) with a hand laceration and head trauma. He was brought in by emergency medical services, who reported he fell while intoxicated, sustained a hand laceration, and was then punched in the face by an unknown bystander who reported he had been somnolent and bleeding on his property. The patient reported a relapse in which he used a bag of fentanyl by unknown route prior to these events. He denied any pain during nurse triage. On arrival to the ED, he was somnolent. He was noted to have dry mucous membranes, a small left thumb laceration, pinpoint pupils, scattered scalp ecchymoses, and a left knee abrasion. Thorough exam revealed no extremity deformity or tenderness. He awakened to command and answered basic questions but fell asleep quickly. Initial history was limited by opioid intoxication, but he denied any significant pain during history or physical exam.

His initial vital signs were as follows: heart rate 126 beats per minute; blood pressure 93/51 millimeters of mercury, respirations 12 breaths per minute, temperature 98.2 °F (36.8 °C), and an oxygen saturation of 85% on room air. His glucose fingerstick was 116 milligrams per deciliter (mg/dL) (reference range: 60–100 mg/dL). The patient was placed on nasal cannula with prompt resolution of hypoxia, and his vital signs normalized without other intervention during a period of observation.

Approximately three and a half hours after arrival, the patient was fully alert and oriented, and he began complaining of pain to his left hip as well as his neck and waist. He was unable to stand upright to ambulate. His repeat physical exam was now notable for firm, tense thoracic and lumbar paraspinal musculature, worse on his left side. At this point because there was a clinical concern for the development of paraspinal compartment syndrome, additional labs and imaging were ordered. The initial labs were remarkable for leukocytosis of 14.5 x 10^3^ per cubic millimeter (K/mm^3^) (4.0–11.0 K/mm^3^), creatinine 2.50 mg/dL (0.80–1.30 mg/dL) (three months prior 1.11 mg/dL), potassium 3.0 millimoles per liter (mmol/L) (3.5–5.2 mmol/L), aspartate aminotransferase 236 units per liter (U/L) (0–34 U/L), alanine aminotransferase 65 U/L (0–44 U/L), anion gap 19 mmol/L (6–16 mmol/L), and creatinine kinase (CK) 19,323 U/L (49–174 U/L). Urine drug screen was positive for benzodiazepines, cocaine, opiates, and fentanyl. A computed tomography (CT) head and cervical spine showed severe sinusitis without acute trauma. Chest radiograph showed slight prominent interstitial markings with patchy areas of reticular nodular densities. A CT abdomen and pelvis showed ground-glass opacities at the lung bases without other acute abnormalities, and no findings were reported related to the paraspinal musculature.

The patient was transferred to the main academic ED for evaluation by the general surgery team, a specialty not available at the community site where the patient presented. He had a repeat CK level that was increasingly elevated at 29,025 U/L despite receiving two liters of normal saline. Based on the physical exam and rising CK, he was diagnosed with paraspinal compartment syndrome and taken to the operating room for bilateral paraspinal musculature fasciotomies. Compartment pressures were not measured as to not delay the necessary surgical intervention. In the operating room, the erector spinae were noted to be bulging, but reactive and viable. The CK peaked at 103,310 U/L and then began down-trending.

On hospital day five, the patient had hemodialysis for rising creatinine (10.10 mg/dL) and hyperkalemia (5.7 mmol/L). On hospital day eight, he underwent fasciotomy closure. He had improving kidney function and did not require further hemodialysis. He was discharged to a recovery house on hospital day 13. Creatinine at time of discharge was 1.53 mg/dL. After discharge, the patient had multiple outpatient follow-up visits with trauma surgery and sports medicine. He was noted to have persistent low back stiffness, but no issues with ambulation or functional limitations.

*CPC-EM Capsule*

What do we already know about this clinical entity?

*Paraspinal is a rare location for compartment syndrome and is typically related to exercise. Delay in diagnosis can worsen patient clinical outcomes*.

What makes this presentation of disease reportable?

*This is a unique presentation of acute paraspinal compartment syndrome and is only the second case documented of paraspinal compartment syndrome related to opioid use*.

What is the major learning point?

*Clinicians should assess all major compartments in patients with history of illicit opioid use and have high index of suspicion for paraspinal compartment syndrome*.

How might this improve emergency medicine practice?

*Raising the index of suspicion for acute paraspinal compartment syndrome in patients using illicit opiates can help reduce the morbidity and mortality in a vulnerable population*.

## DISCUSSION

Functional groups of muscle fibers and their associated neurovasculature are organized and contained by layers of connective tissue known as fascia. Collectively, these units are known as compartments. Acute compartment syndrome is an emergent condition where the pressure within the muscle compartments is greater than the perfusion pressure of the tissue. It is most often identified as a condition of the extremities. Paraspinal compartment syndrome was first described in the literature in 1985.^6^ Along the dorsal aspect of the thorax are columns of muscle fibers contained by the thoracolumbar fascia. When localized inflammation occurs, muscle fibers swell within the immobile surrounding fascia. This restricted expansion leads to an increase in compartment pressures, which if not released leads to compartment syndrome and may lead to muscle ischemia, necrosis, and subsequent organ dysfunction such as renal failure.

A case report and review of the literature from 2022 found 37 cases of paraspinal compartment syndrome. Most cases (67.5%) were related to exertion or weightlifting. Another 16.2% were related to surgery. Of the 37 cases, 29 received magnetic resonance imaging (MRI). While MRI may show changes in the paraspinal muscles such as edema, decreased perfusion, or necrosis, it is not necessary for diagnosis and may further delay treatment. Patients who were treated operatively were significantly more likely to be pain-free or return to baseline activities compared to patients treated non-operatively.^5^

In 2020, a case of paraspinal compartment syndrome was reported in a patient with seizures and altered mental status. The patient’s urine drug screening was positive for cocaine and fentanyl. To our knowledge, this is the only other case described in the literature of paraspinal compartment syndrome related to fentanyl use.^7^ Rhabdomyolysis has been described in the context of heroin use previously with the suggestion that prolonged immobilization and pressure injuries while intoxicated are leading factors. Heroin-related compartment syndrome has been described with increasing frequency, secondary to tissue injury from prolonged immobilization in settings of intoxication and obtundation. The gluteal compartment has been the most common location identified in the literature.^8^ A phenomenon associated with fentanyl use specifically is wooden chest syndrome, a condition of chest wall and extremity muscle rigidity leading to rapid respiratory depression and obtundation. This syndrome has been uniquely identified with synthetic opiates such as fentanyl, is seen less with heroin and other opiates,^9^ and may contribute to the development of compartment syndrome.

Xylazine, commonly referred to as “tranq,” is an increasingly recognized adulterant in the illicit drug supply in Philadelphia. Xylazine is a veterinary anesthetic and analgesic and is an alpha-2 adrenergic agonist that has synergistic effects when used with fentanyl. It is known to cause profound sedation and prolong the effects of fentanyl. Ninety-one percent of fentanyl or heroin samples from 2021 contained xylazine.^10^ Testing from 2022 revealed increasing xylazine presence in the city drug supply and per the Philadelphia Department of Public Health, “people who use illicit opioids in Philadelphia are almost certainly being exposed to xylazine.” Fatal overdoses involving xylazine from 2015–2021 increased from 15 to 434.^11^ We theorize that the increased sedation and prolonging of fentanyl effects may also increase the risk for the development of compartment syndrome in the setting of prolonged immobilization.

## CONCLUSION

Acute paraspinal compartment syndrome is an uncommon but high-stakes condition. This case demonstrates that paraspinal compartment syndrome may be associated with fentanyl and possibly xylazine use due to their profound and prolonged sedative affects. With the increase in synthetic opioids and associated adulterants, patients who use these substances may be at increased risk for this condition. Given that patients may present acutely intoxicated, limiting a clinician’s ability to obtain suggestive history, it is imperative that the clinician recognize the signs of paraspinal compartment syndrome and maintain a high index of suspicion to initiate definitive care and reduce risk for acute and long-term complications.
